# *N*-Terminally Lipidated Sialorphin Analogs—Synthesis, Molecular Modeling, In Vitro Effect on Enkephalins Degradation by NEP and Treatment of Intestinal Inflammation in Mice

**DOI:** 10.3390/ijms232214450

**Published:** 2022-11-21

**Authors:** Małgorzata Sobocińska, Jakub Fichna, Artur Giełdoń, Piotr Skowron, Elżbieta Kamysz

**Affiliations:** 1 Laboratory of Chemistry of Biological Macromolecules, Department of Molecular Biotechnology, Faculty of Chemistry, University of Gdansk, 80-308 Gdansk, Poland; 2 Department of Biochemistry, Faculty of Medicine, Medical University of Lodz, 92-215 Lodz, Poland; 3 Laboratory of Simulation of Polymers, Department of Theoretical Chemistry, Faculty of Chemistry, University of Gdansk, 80-308 Gdansk, Poland; 4 Laboratory of Bionanotechnology, Department of Molecular Biotechnology, Faculty of Chemistry, University of Gdansk, 80-308 Gdansk, Poland

**Keywords:** enkephalins, sialorphin, neutral endopeptidase, peptides synthesis, molecular modelling, structure–activity relationship

## Abstract

Pharmacotherapy for inflammatory bowel disease (IBD) is difficult, and some patients do not respond to currently available treatments. Therefore, the discovery of novel anti-IBD agents is imperative. Our aim was the synthesis of lipidated analogs of sialorphin and the in vitro characterization of their effect on the degradation of Met-enkephalin by neutral endopeptidase (NEP). We also investigated in vivo whether the most active inhibitor (peptide **VIII**) selected in the in vitro studies could be a potential candidate for the treatment of colitis. Peptides were synthesized by the solid-phase method. Molecular modeling technique was used to explain the effect of fatty acid chain length in sialorphin analogs on the ligand–enzyme interactions. The anti-inflammatory effect was evaluated in the dextran sulphate sodium (DSS)-induced model of colitis in mice. Peptide **VIII** containing stearic acid turned out to be in vitro the strongest inhibitor of NEP. We have also shown that the length of the chain of stearic acid fits the size of the grove of NEP. Peptides **VII** and **VIII** exhibited in vivo similar anti-inflammatory activity. Our results suggest that lipidation of sialorphin molecule is a promising direction in the search for NEP inhibitors that protect enkephalins.

## 1. Introduction

Inflammatory bowel diseases (IBD) are idiopathic disorders that involve chronic, relapsing inflammation of the gastrointestinal (GI) tract in the pathogenesis of which genetic, immunological, and environmental factors play a role. The most common types of IBD include Crohn’s disease and ulcerative colitis. IBDs affect mostly patients between the ages of 15 and 30 years; however, IBD may occur at any age. Based on the latest report of the European Crohn’s and Colitis Organization-Epidemiological Committee (ECCO-EpiCom) the mean annual incidence rate of IBD is 11.3/100,000 in Eastern Europe and 14.0/100,000 in Western Europe [1]. IBD is probably caused by industrialization, improved socioeconomic status, as well as dietary and lifestyle changes [2]. Importantly, the pharmacological treatment of IBD, which includes 5-aminosalicylates, immunosuppressive agents, corticosteroids, and biological therapeutics, is complex and not always effective, particularly in the case of biologics, while side effects may be substantial. Moreover, there are high economic costs of IBD treatment [3,4]. Colitis-associated colorectal cancer (CAC) remains a critical complication of ulcerative colitis (UC) with a mortality of approximately 15% [5]. Crohn’s disease is associated with an increased risk of colorectal carcinoma. Alopecia areata may be a side effect of biological therapy to treat IBD with anti-TNF-α agents [6]. These facts, together with increasing comorbidities in patients with IBD, imply that urgent, more complex, and novel therapeutic strategies in the treatment of IBD are needed.

One of the latest strategies proposed in IBD pharmacotherapy is using neprilysin (NEP) inhibitors to protect the endogenous opioids, enkephalins (Met- and Leu-enkephalin)—which participate in antinociception [7], the regulation of GI motility [7], the modulation of the immune system [8,9], affecting anti-inflammatory, hormonal, and behavioral responses [10]—against fast degradation and loss of activity. 

Enkephalins are produced by the pituitary, brain, and adrenal glands; sympathetic nervous system; pancreas; and are also formed in the GI tract in gastric and intestinal endocrine cells [11,12]. Numerous investigations indicate the participation of Met-enkephalin and other opioids in regulating intestinal motility, in gastric, pancreatic, and intestinal secretion and in carbohydrate metabolism [13,14]. Met- and Leu-enkephalins were also found to be synthesized in leukocytes and may thus participate in inflammatory response [11]. Both peptides are potent DOR agonists, and additionally possess some affinity at MOR. The investigations carried out in mouse models have confirmed the presence of opioid receptors on the surface of leukocytes and their ability to synthesize and secrete opioid peptides following appropriate stimulation [15]. Owczarek et al. demonstrated that changes in Met-enkephalin levels in CD and UC may play a role in the pathogenesis as well as the course of these diseases [11]. It was found that the serum levels of Met-enkephalin were decreased in patients with IBD in comparison to healthy volunteers. Moreover, higher levels of Met-enkephalin were found in colonic biopsies collected from inflammatory lesions from patients with IBD, compared to biopsies from non-inflamed lesions. [11,12]. Enkephalins exert an anti-inflammatory effect through modulating the immune system and inflammatory response and controlling hormone levels in both IBD patients and animal models of IBD [11]. These peptides are a link between the neuroendocrine and immune systems, and the immunomodulatory effect of enkephalins may play a significant clinical role in immune-mediated diseases [11].

Neprilysin, also called neutral endopeptidase, enkephalinase, or atriopeptidase, is a zinc-dependent peptidase localized in the plasma membrane with the catalytic site facing the extracellular space and belonging to the M13 family. It is the major enzyme involved in the metabolic inactivation of a variety of bioactive peptides including—as mentioned above—the enkephalins as well substance P, endothelin, bradykinin, atrial natriuretic factor, and amyloid-β (Aβ) [16]. NEP inhibitors are already used as a new class of drugs to treat high blood pressure and heart failure. They work by blocking the action of NEP, thus preventing the breakdown of natriuretic peptides [17]. Semenogelin-1-derived peptide (RSIY-11) is another potent competitive inhibitor of NEP; when added to seminal fluid, RSIY-11 significantly increases sperm motility [18]. Inhibition of NEP is also studied as a therapeutic target for the treatment of kidney disease [19]. There is evidence that NEP has protective activity against Alzheimer’s disease (AD) by degrading the Aβ peptide. Inhibition of NEP in mice has resulted in a large increase in the levels of Aβ and plaque-like deposits in the brain, which may lead to cognitive impairment [20,21]. 

NEPs are widely distributed in the human body and are significantly involved in physiological modulation and pathophysiological processes in the GI tract. The indirect stimulation of opioid receptors by the blockade of NEP is a promising pharmacological strategy for the treatment of IBD and may become of greater importance than the use of classical opioid agonists. Among endogenous inhibitors of NEP, there is sialorphin (QHNPR). Studies have confirmed its efficacy in blocking the activity of NEP in vitro and in vivo in animal models. It has been demonstrated that this inhibitor possesses an anti-inflammatory effect either directly or indirectly by affecting the level of enkephalins [22]. The significant limitation on the use of sialorphin in systemic therapeutic applications is its sensitivity to degradation in human plasma.

In the present work we reported the synthesis of a series of lipopeptides composed of lipophilic moieties, i.e., saturated fatty acids residues (C_12_–C_18_), covalently attached to the *N*-terminus of sialorphin via a peptidic linker consisting of lysine residues. We also characterized in vitro the effect of obtained sialorphin analogs on the degradation of Met-enkephalin by NEP and investigated the stability of the most active inhibitors in the human plasma. The fatty acid tail was introduced to the sialorphin molecule, because in our earlier studies it was found that addition of the palmitic acid residue (Palm-) by a dipeptide Lys-Lys linker resulted in a compound that was a potent inhibitor of Leu-enkephalin degradation by NEP and significantly attenuated TNBS-induced colitis in mice [23].

We also used molecular modeling technique to explain the influence of fatty acid chain length in sialorphin analogs on the ligand–enzyme interactions and investigated in vivo whether the most active inhibitor selected in the in vitro studies could be a potential candidate for the treatment of intestinal inflammation.

## 2. Results and Discussion

### 2.1. Peptides Synthesis and Purification

Sialorphin and its seven analogs (peptides **II**–**VIII**) modified in the *N*-terminal part with one, two, and three lysine residues and conjugated with different lipopeptides: Laur-Lys-Lys, Mir-Lys-Lys, Palm-Lys-Lys, and Stear-Lys-Lys were synthesized using the solid-phase method [23,24]. The peptides’ purity after HPLC purification was higher than 98%, as determined by analytical HPLC. The MALDI-TOF mass spectrometry confirmed the identity of the purified peptides. The sequences and physicochemical characteristics of peptides **I**–**VIII** are shown in Table 1.

### 2.2. Effect of Sialorphin and Its Analogs on Degradation of Met-Enkephalin by NEP 

The results of the in vitro investigation demonstrated that conjugation of the *N*-terminal part of sialorphin with a short lipopeptide, Stear-Lys-Lys, resulted in compound **VIII** that was the most potent inhibitor of Met-enkephalin degradation by NEP (Table 2). The conjugation of the *N*-terminal part of sialorphin with a short lipopeptide Mir-Lys-Lys and Laur-Lys-Lys gave two inhibitors (peptides **V** and **VI**) that increased the half-lives (t_1/2_) of Met-enkephalin less potently than peptides **VII** and **VIII**.

The designed sialorphin analogs modified at the *N*-terminus with one, two, and three lysine residues (peptides **II**–**IV**) showed comparable inhibitory activity (half-lives 90 ± 3, 96 ± 2 and 83 ± 1 min), only slightly higher than the parent structure ( half-live 78 ± 2 min), with the most active analog containing two lysine residues at the *N*-terminus (peptide **III**).

The attachment of various short lipopeptides to the *N*-terminus of the sialorphin molecule (peptides **V**–**VIII**) showed a correlation between the hydrophobicity and the inhibitory activity of the obtained analogs. As the length of the hydrocarbon chain forming the fatty acid structure increased, the inhibitory activity of the peptides **V**–**VIII** increased. The peptide **VIII** containing stearic acid at the *N*-terminus turned out to be the strongest inhibitor NEP from this group.

### 2.3. Stability in the Human Plasma

Compounds **VII** and **VIII** were the strongest inhibitors of NEP in vitro and were thus included in stability assays. Peptides were incubated in the human plasma at 37 °C, and their degradation was analyzed by RP HPLC. The stability of peptides **VII**–**VIII** was compared to that of a heterodimer of sialorphin and spinorphin and not sialorphin alone. In previously described stability assays of sialorphin alone, the 48% TFA solution was used to precipitate proteins before HPLC analysis [25]. In the case of the peptides **VII** and **VIII,** we had to use ethanol instead of the TFA solution. This change was required by the fact that in the latter solution, an emergence of a degradation product that was characterized by identical retention time on HPLC as that of peptides **VII** and **VIII** occurred. Consequently, a different reference was used (heterodimer of sialorphin and spinorphin instead of sialorphin).

**Figure 1:**
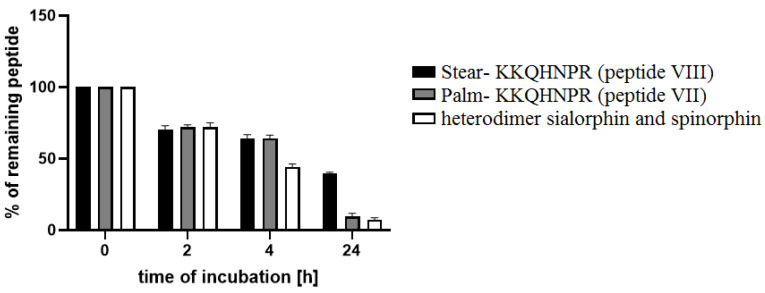
Conjugates sialorphin with a short lipopeptides: Palm-Lys-Lys (peptide **VII**) and Stear-Lys-Lys (peptide **VIII**) and heterodimer sialorphin and spinorphin stabilities in human plasma. The means of the % of remaining peptide with a standard deviation are presented.

As in the case of the inhibitory activity, the stability of peptides **VII** and **VIII** in the plasma was greater with the increase in the length of the hydrocarbon chain forming the fatty acid structure. As shown in Figure 1, after 4 h of incubation, analogs with attached short lipopeptide (**VII** and **VIII**) displayed comparable stability in plasma, yet 1.4 times greater than did the heterodimer. After 24 h of incubation, the levels of peptides **VII** and **VIII** were 1.3 and 5.5 times higher, respectively, in relation to the heterodimer.

We also can conclude based on the current results of the stability assays (with the method using ethanol) and the results of the stability studies of sialorphin and heterodimer sialorphin and spinorphin (method using 48% TFA solution) described previously [25], that the stability of peptides **VII** and **VIII** in the human plasma was much greater than sialorphin.

### 2.4. Molecular Modeling

**Figure 2:**
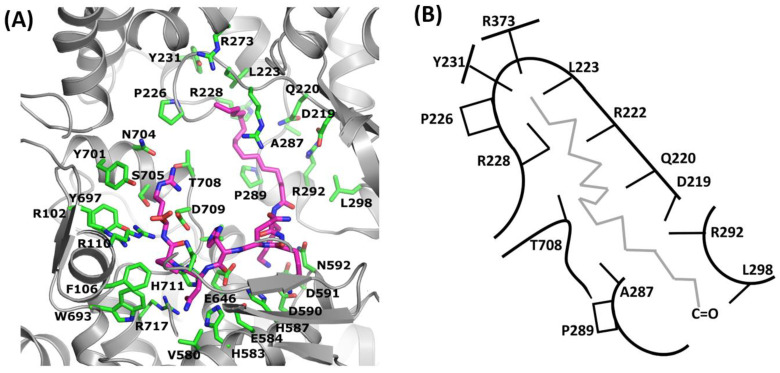
The result of the docking simulation of Stear-KKQHNPR ligand to human NEP structure. (**A**) Protein shape was colored gray. The interacting residues were described with one letter abbreviation and colored green. The ligand was colored purple. (**B**) Schematic representation of the fatty acid aliphatic chain binding pocket. One letter abbreviation was used to point to the interacting residues. The location (direction) of the sidechains constituting the binding pocket was shown as sticks.

The detailed analysis of the degradation rates of the Met-enkephalin incubated with neutral endopeptidase with a presence of peptides **III** and **V**–**VIII** shows unexpected results. The peptide **III**, without an aliphatic chain, shows high inhibition rate (see Table 2). However, the addition of lauric and myristic acid residue with 12 and 14 carbon atoms, respectively, at the *N*-terminal part of the peptide, caused an abolition of the inhibition rate of those peptides. On the contrary, the addition of palmitic (C_16_) and stearic (C_18_) acid increased the inhibition rate by two and almost eight times, respectively (see Table 2). Therefore, the question was how the small change of the length of the aliphatic chain from (C_14_) to (C_16_) changes the inhibition rate so dramatically. To answer this question we performed a series of docking simulations by using PLANTS software. In the unrestrained docking simulation, most of the ligands were located in the grove composed of D219, Q220, R222, L223, P226, R228, A287, R292, and L298 and located above the experimentally found binding pocket. Since the ligand was not located as shown by the experimental data, we performed a second docking experiment with a distance restraint defined on asparagine residue. This residue should be located in the S1′ binding pocket. As expected, the second docking experiment was much more consistent with experiment, since the backbone of the ligand was located in the same place as I20 inhibitor in 2QPJ structure. This time the aliphatic chain was located in the binding grove described above (Figure 2). We did not expect such a result, since most of the pointed residues are hydrophilic. The detailed analysis of the obtained model allowed us to explain this. The aliphatic chain of the fatty acid attached to the *N*-terminal part of the inhibitor interacts with the aliphatic part of the R222, R228, and R292 sidechains. The guanidine group is directed opposite. As a consequence, the natural contacts of those residues are being preserved. 

The protein–fatty acid interactions are mostly via charged head [26]; however, in this case, the aliphatic chain plays a crucial role. The location of the aliphatic chain of the fatty acid in the pointed binding grove does not explain the tremendous difference between (C_14_) and (C_16_). If we compare the size of the grove with the length of the chain of the fatty acid, it appeared that (C_18_) fits perfectly. When the chain is too small (C_14_) and smaller, we may suspect that the interaction with the protein is not strong enough to keep it stable. When the aliphatic chain of the fatty acid is not “anchored” in the protein binding pocket, it can move “freely”. As a consequence, it can pull out the inhibitor from the protein binding pocket, and we could not observe the inhibition activity (see Table 2). The interactions of the protein with the aliphatic chain are weak. We may suspect that it will remain stable when it will “fit well” into the binding pocket, and the number of interactions will be large enough to keep the chain stable [27]. 

### 2.5. Peptides **VII** and **VIII** Alleviate Inflammation in the DSS-Induced Mouse Model of Colitis

As a next step, we examined the effect of peptides in the mouse model of DSS-induced experimental colitis, which mimics the symptoms of ulcerative colitis in humans. Both peptides were tested in the same dose (1 mg/kg, twice daily, i.p.).

**Figure 3:**
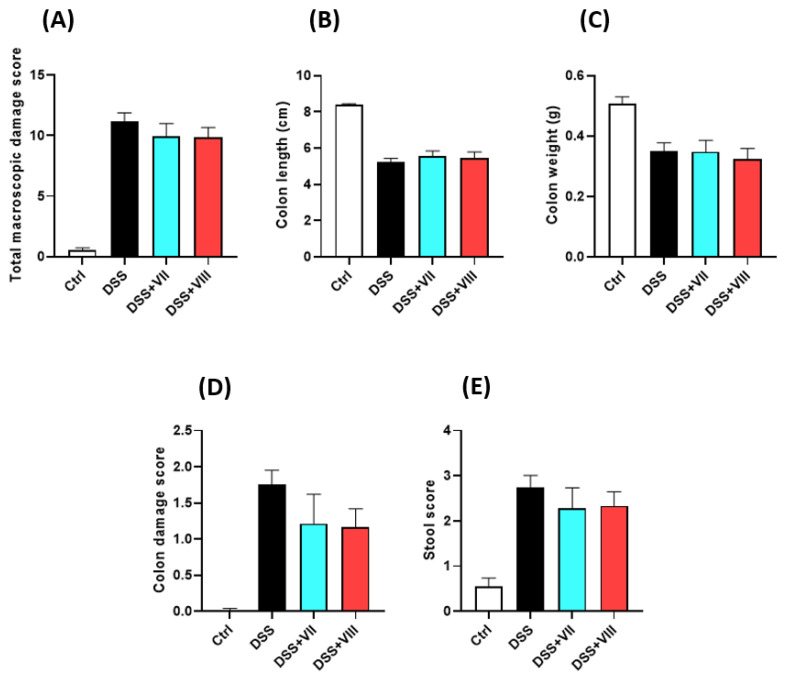
The effect of the peptides on colonic inflammation in DSS-induced mouse model of colitis: total macroscopic damage score (**A**), colon length (**B**), colon weight (**C**), colon damage score (**D**), and stool score (**E**) for control, DSS-only treated mice, and mice with DSS-induced colitis treated with 1 mg/kg peptide **VII** or peptide **VIII** twice daily. Data represent mean ± SEM of *n* = 5–10 animals per group.

The total macroscopic damage score was significantly increased when the mice were treated with DSS (0.55 ± 0.18 for control vs. 11.20 ± 0.60 for DSS-only treated mice, *p* < 0.001, Figure 3). The administration of peptides caused a non-significant decrease in the total macroscopic damage score in comparison to DSS-only treated animals (10.20 ± 0.99, for **VII**; 9.70 ± 1.45, *p* < 0.05 for **VIII**). Similar tendency suggesting the anti-inflammatory effect of the tested peptides was observed in colon damage score and stool score.

**Figure 4:**
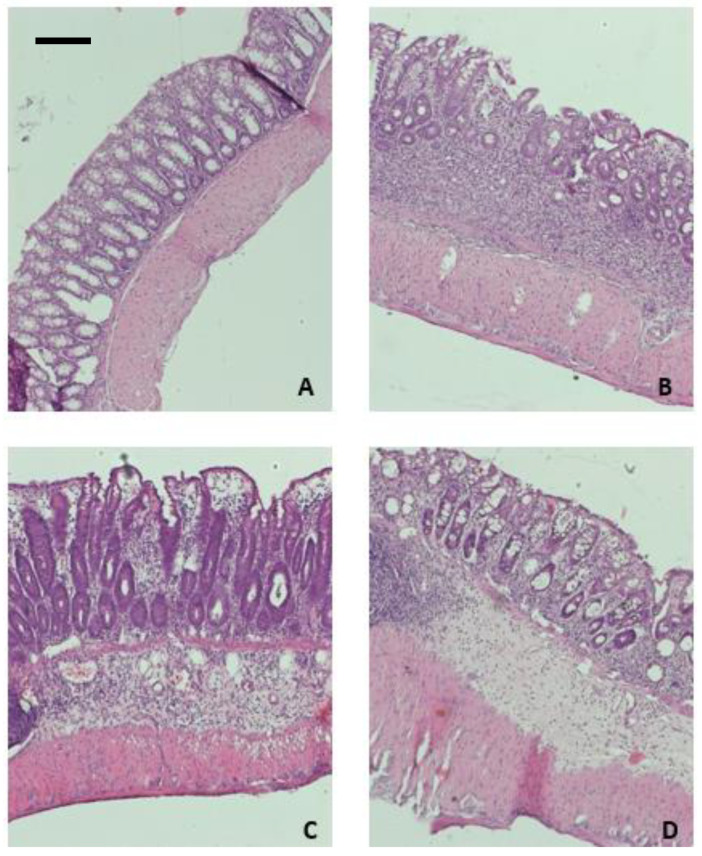
Hematoxylin and eosin staining of mouse colon samples: control (**A**), DSS (**B**), DSS + peptide **VII** (**C**), and DSS + peptide **VIII** (**D**). Scale bar = 100 μm.

The parameters considered to determine the microscopic damage score included the presence of intact goblet cells, crypt morphology, mucosal architecture, muscle thickness, and cell infiltration. The examination of collected samples revealed a significantly increased score for DSS-treated animals (Figure 4). Treatment with peptide **VII** significantly improved colon architecture, with reversal of crypt morphology damage and decrease of cell infiltration. The effect of peptide **VIII** on microscopic score was less pronounced.

## 3. Materials and Methods

### 3.1. Peptides Synthesis and Purification

All peptides were synthesized manually by the solid phase approach on 2-chlorotrityl chloride resin (loading 0.3–0.9 mmol/g, 1% DVB, 100–200 mesh, Orpegen Peptide Chemicals GmbH, Heidelberg, Germany) using Fmoc (9-fluorenylmethoxycarbonyl) chemistry [23,24]. N^α^-Fmoc-protected amino acids, stearic acid, palmitic acid, myristic acid, lauric acid, and the reagents used for the solid-phase synthesis were obtained from Iris Biotech GmbH (Marktredwitz, Germany). The following amino acid derivatives were used: Fmoc-Gln(Trt), Fmoc-His(Trt), Fmoc-Asn(Trt), Fmoc-Pro, Fmoc-Arg(Pbf), Fmoc-Lys(Boc). The first amino acid was bound to the resin according to Barlos et al. with a loading dose of 0.7 mmol/g [28]. Fully protected peptide resins were synthesized according to standard procedures involving (i) deprotection steps using a 20% solution of piperidine in *N,N*-dimethylformamide (DMF), for 5 and 15 min and (ii) coupling reactions carried out with a three-fold molar excess of the protected amino acid (Fmoc-AA) dissolved in DMF/dichloromethane (DCM) with addition of 1-hydroxybenzotriazole (HOBt) and *N*,*N’*-diisopropylcarbodiimide (DIC) for 2 h (Fmoc-AA:HOBt:DIC; 1:1:1:1). The completeness of each coupling reaction was monitored by the chloranil test. After completion of the synthesis, the protected peptidyl resins were treated with trifluoroacetic acid (TFA)/triisopropylsilane (TIS)/H_2_O (95.5: 2.5: 2.5; *v/v/v*) and stirred for 2 h [23,24]. Then the peptides were precipitated with cold diethyl ether to afford crude products. The resulting materials were dissolved in water, frozen, and lyophilized. 

The crude material obtained in this way was purified by the Reverse Phase High Performance Liquid Chromatography (RP-HPLC) on Kromasil C8 column (8 × 250 mm, 100-Å pore size, 5-μm particle size) with linear gradient 2–40% of [B] in [A] for 30 min (QHNPR, KQHNPR, KKQHNPR and KKKQHNPR), linear gradient 40–80% of [B] in [A] for 60 min (Stear-KKQHNPR), linear gradient 20–80% of [B] in [A] for 40 min (Pal-KKQHNPR), linear gradient 10–60% of [B] in [A] for 35 min (Mir-KKQHNPR), and linear gradient 20–70% of [B] in [A] for 50 min (Laur-KKQHNPR), where [A] is 0.1% TFA in water and [B] is 0.1% TFA in acetonitrile, with a flow rate of 10 mL/min. The eluates were fractionated and analyzed by the analytical RP-HPLC. The purity of the peptides was checked on a Beckman HPLC controlled by the Lp-Chrom system. Fractions containing the pure peptides (>98%) were pooled and lyophilized. The mass spectrometry analysis of the synthesized compounds were carried out on a matrix-assisted laser desorption/ionization mass spectrometry (a Biflex III MALDI-TOF instrument, Bruker Daltonics, Karlsruhe, Germany) using an α-cyano-4-hydroxycinnamic acid matrix. 

### 3.2. Determination of Met-Enkephalin Degradation Rates

Determination of Met-enkephalin degradation rates was performed, as described previously [29]. Purified enzyme NEP was purchased from Merck (Warsaw, Poland). Solutions of Met-enkephalin, NEP, and inhibitors were prepared by dissolving them in a Tris–HCl buffer (50 mM, pH 7.4). In investigated samples, the concentrations of Met-enkephalin, inhibitors, and NEP were, respectively, 0.0413 mM, 0.156 mM, and 5.687 nM. The samples consisting of NEP, Met-enkephalin, and the inhibitor were incubated over 0, 30, 60, 90, and 120 min at 37 °C in a final volume of 300 μL. The reaction was stopped with a 1 M aqueous HCl solution. In the next steps, the samples were centrifuged and the supernatants were filtered and analyzed by RP-HPLC. All measurements were performed in triplicates.

### 3.3. Stability Peptides **VII** and **VIII** in Human Plasma

Plasma-stability assays were performed by applying procedure described by Sobocińska et al. [30]. Each peptide solution was prepared by dissolving 1 mg of a peptide in 1 mL of PBS buffer (pH 7.42). The samples containing the peptide (200 µL) and freshly refrozen plasma (50 µL) were incubated at 37 °C. The samples were analyzed after 0, 2, 4, and 24 h. At each time interval 40 μL of the mixture was taken and deproteinized by addition of a ethanol (120 µL). The aliquots were centrifuged at 14,500 rpm for 5 min. The supernatants were filtered over Millipore Millex-GV syringe filters (Merck Life Science, Poznan, Poland) and analyzed with RP-HPLC on a Phenomenex Gemini-NX C18 5 µm column (4.6 mm × 150 mm) using the solvent system of 0.1% TFA in water (A) and 0.1% TFA in acetonitrile (B) and linear gradients of 20–80% (B) over 20 min, at a flow rate of 1.5 mL/min. To assess the stability at each time interval, the area of the peptide peak from the chromatogram was calculated and expressed as the percentage of the area of the peak recorded just after mixing a peptide with plasma (0-hour). The assay was performed at least in triplicate for each peptide.

### 3.4. Molecular Modeling

Human NEP structure (PDB code 2QPJ) with inhibitor (called I20) [31] and our previous work [23] were used as a template for modeling. Peptide **XIII** (KKQRFSR) from [18] was computer mutated to KKQHNPR. The asparagine sidechain was located in the S1′ binding pocket. Two runs of the docking experiment of peptides **V**–**VIII** by using PLANTS software [32,33] were performed. PLANTS software combines the randomized solution construction by the Ant Colony Optimization algorithm with a local search algorithm. The position of CA atom of asparagine residue was declared as a center of the docking space with 21Å radius. The partial charges of the amino acids were taken from AMBER database [34,35] and partial charges for fatty acid residues were calculated with RESP procedure as implemented in AMBER forcefield. 

The first docking experiment was without any restraints, while the second applied a distance restraint for the position of the asparagine sidechain. The results were analyzed by using RASMOL AB software (Poland, https://etoh.chem.ug.edu.pl/rasmol/) [36].

### 3.5. Animals

Male BALB/C mice obtained from University of Lodz, Poland, weighing 22–25 g (6–8 weeks of age), were used. The mice were kept at a constant temperature (22–23 °C) and maintained under a 12 h light/dark cycle with constant access to laboratory chow and tap water. All animal protocols were approved by the Medical University of Lodz Animal Care Committee (Protocol 2/ŁB 123/2019 and 17/ŁB 132/2019) and complied with Directive 2010/63/EU of the European Parliament and of the Council of 22 September 2010. All efforts were made to minimize animal suffering and to reduce the number of animals used.

#### 3.5.1. Drugs and Reagents

All drugs and reagents, unless stated otherwise, were purchased from Sigma-Aldrich (Poznan, Poland).

#### 3.5.2. Induction of Colitis

Colonic inflammation was induced as described before [37]. From day 0 to day 5, mice received DSS: 4% *w/v* and molecular weight 40,000 (PanReac AppliChem, Darmstadt, Germany) in drinking water. On days 6 and 7, the DSS solution was replaced with tap water. Control animals were given drinking water throughout the whole experiment. Animal body weight was monitored daily. Mice were sacrificed by cervical dislocation on day 7, and colonic damage was evaluated.

#### 3.5.3. Pharmacological Treatments

Treatment was administered intraperitoneally (i.p.) twice daily from day 3 to day 6 after colitis induction, 100 µL/mouse. Peptides were administered at the dose of 1 mg/kg bw. Control and DSS groups received vehicle alone (100 µL/mouse, i.p.).

#### 3.5.4. Evaluation of Colonic Damage (Macroscopic and Microscopic Score Evaluation)

On day 7 of the experiment, mice were euthanized. For total macroscopic damage score evaluation, the colon was removed immediately after cervical dislocation and weighed with fecal content. Next, the colon was opened longitudinally, and the feces were removed. 

Total macroscopic damage score was calculated based on the stool consistency (0—normal, well-shaped fecal pellets; 3—diarrhea), colon epithelial damage (0–3), and colon length and weight scores, calculated as a loss of each parameter relative to the control group (0 points, ≤5% weight/length loss; 1 point, 5–14% weight/length loss; 2 points, 15–24% weight/length loss; 3 points, 25–35% weight/length loss; and 4 points, ≥35% weight/length loss). Score = 0 means no inflammation. The presence (score = 1) or absence (score = 0) of fecal blood was also recorded. The macroscopic scoring was performed in a blind manner.

Distal colon segments (approx. 0.5 cm in length) were stapled flat, mucosal side up, onto cardboard and fixed in 10% neutral-buffered formalin for at least 24 h at 4 °C. Next, samples were dehydrated in sucrose, embedded in paraffin, sectioned at 5 μm, and mounted onto slides. The hematoxylin-eosin-stained sections were examined using a Zeiss Axio Imager setup (Jena, Germany). Microscopic total damage score was determined based on the following parameters: presence (score = 1) or absence (score = 0) of goblet cell depletion, the presence (score = 1) or absence (score = 0) of crypt abscesses, the destruction of mucosal architecture (normal = 1, moderate = 2, extensive = 3), the extent of muscle thickening (normal = 1, moderate = 2, extensive = 3), and the presence and degree of immune cell infiltration (normal = 1, moderate = 2, transmural = 3).

#### 3.5.5. Statistics

Statistical analysis was performed utilizing Prism 9.0.1 software (GraphPad Software Inc., La Jolla, CA, USA). The data are expressed as means  ±  SEM. The Shapiro–Wilk test was used to confirm the Gaussian distributions of raw data. One-way analysis of variance (ANOVA) followed by a Newman–Keuls post-hoc test were used for testing the differences among means. *p* values  <  0.05 were considered as statistically significant.

## 4. Conclusions

The pathogenesis of IBD is still not completely understood. Gut dysfunction in patients with active IBD may be connected with changes in many factors, for instance increased prostaglandin levels as a result of COX-2 overexpression in neural cells of the myenteric plexus or decreased opioid peptides level rapidly degraded by endogenous enzymes (NEP, APN, DPPIII, and ACE). 

Here authors demonstrated that the lipidated analogs of sialorphin (peptides VII [23] and VIII) in vitro are strong inhibitors of NEP and attenuate DSS-induced colitis in mice. The results of the in vitro studies clearly demonstrated that the length of the carbohydrogen chain of a fatty acid of short lipopeptide conjugated to sialorphin is crucial for protection of Met-enkephalin against degradation by NEP. Moreover, lipidation of the NEP inhibitor (peptides VII and VIII) increased the stability of investigated peptides in the human plasma, and the increase in the stability was greater with the increase in the length of the hydrocarbon chain forming the fatty acid structure. The pharmacological profile of peptide VIII in vitro was better in comparison to peptide VII, but the anti-inflammatory activity of peptide VII in vivo was similar to peptide VIII when macroscopic and microscopic scores were compared. The observed effect suggests that the PD/PK profile of obtained peptides is equally important when comparing their efficacy.

Summing up, our results suggest that lipidation of the sialorphin molecule is a promising direction in the search for NEP inhibitors that protect enkephalins. Moreover, the opioid system plays a critical role in maintaining GI homeostasis, and, because of that, targeting of the endogenous opioid system with NEP inhibitors may be a promising therapeutic strategy for the treatment of IBD. Development of new compounds with longer half-life is important to decrease the number of injections and improve patients’ acceptance. Our findings will provide data for future clinical research in the treatment of IBD.

## Figures and Tables

**Table 1:** Physicochemical properties of peptides **I**–**VIII**.

Peptide	Sequence	HPLC t_R_ [min]	Molecular Ion	
			calc. [M]^+^	Found [M+H]^+^
**I**	QHNPR	5.5 ^a^	650.3	651.4
**II**	KQHNPR	5.6 ^a^	778.7	779.7
**III**	KKQHNPR	5.5 ^a^	906.5	907.7
**IV**	KKKQHNPR	7.9 ^f^	1034.6	1036.0
**V**	Laur-KKQHNPR	9.5 ^e^	1088.8	1089.9
**VI**	Mir-KKQHNPR	11.5 ^d^	1116.9	1117.9
**VII**	Palm-KKQHNPR	9.1 ^c^	1144.7	1146.3
**VIII**	Stear-KKQHNPR	7.8 ^b^	1172.9	1174.3

^a^ Linear gradient from 2 to 40% of [B] for 15 min, flow rate of 1.5 mL/min; ^b^ linear gradient from 40 to 80% of [B] for 15 min, flow rate of 1.5 mL/min; ^c^ linear gradient from 10 to 80% of [B] for 15 min, flow rate of 1.5 mL/min; ^d^ linear gradient from 10 to 70% of [B] for 15 min, flow rate of 1.5 mL/min; ^e^ linear gradient from 20 to 80% of [B] for 15 min, flow rate of 1.5 mL/min; ^f^ linear gradient from 2 to 40% of [B] for 15 min, flow rate of 1 mL/min.

**Table 2:** Degradation rates (k) and half-lives (t_1/2_) of Met-enkephalin incubated with neutral endopeptidase alone and in the presence of inhibitors.

No	Inhibitor	Met-Enkephalin	
		1000 × k [1/min]	t_1/2_ [min]
**-**	without inhibitor	25.35 ± 1.05	27 ± 1
**I**	QHNPR	8.84 ± 0.27 ***	78 ± 2 ***
**II**	KQHNPR	7.63 ± 0.22 ***	90 ± 3 ***
**III**	KKQHNPR	7.17 ± 0.16 ***	96 ± 2 ***
**IV**	KKKQHNPR	8.28 ± 0.11 ***	83 ± 1 ***
**V**	Laur-KKQHNPR	23.20 ± 0.89	30 ± 1
**VI**	Mir-KKQHNPR	20.06 ± 1.34 **	35 ± 2
**VII**	Palm-KKQHNPR	3.46 ± 0.40 ***	199 ± 2 ***
**VIII**	Stear-KKQHNPR	0.89 ± 0.10 ***	724 ± 43 ***

Data are mean ± SEM. ** *p* < 0.01, *** *p* < 0.001, compared to Met-enkephalin alone.

## Data Availability

Data available, upon reasonable request, from the corresponding author.

## Abbreviations

Boc: tert-butyloxycarbonyl; DCM: dichloromethane; DIC: *N,N*′-diisopropylcarbodiimide; DMF: *N,N*-dimethylformamide; DSS: dextran sulphate sodium; Fmoc: 9-fluorenylmethoxycarbonyl; HOBt: 1-hydroxybenzotriazole; MALDI-TOF MS: matrix-assisted laser desorption/ionization time-of-flight mass spectrometry; NEP: neutral endopeptidase; Pbf: 2,2,4,6,7-pentamethyldihydrobenzofuran-5-sulfonyl residue; RP-HPLC: reversed-phase high-performance liquid chromatography; TFA: trifluoroacetic acid; TIS: triisopropylsilane; Trt: trityl.
